# Employment Status Following Heart Transplantation: Data From the Danish Nationwide Social Service Payment Register During 20 years

**DOI:** 10.3389/ti.2024.12230

**Published:** 2024-04-17

**Authors:** Rikke Elmose Mols, Brian Bridal Løgstrup, István Bakos, Erzsébet Horváth-Puhó, Finn Gustafsson, Hans Eiskjær

**Affiliations:** ^1^ Department of Clinical Medicine, Aarhus University, Aarhus, Denmark; ^2^ Department of Cardiology, Aarhus University Hospital, Aarhus, Denmark; ^3^ Department of Clinical Epidemiology, Aarhus University Hospital and Aarhus University, Aarhus, Denmark; ^4^ Department of Clinical Medicine, University of Copenhagen, Copenhagen, Denmark; ^5^ Department of Cardiology, University Hospital of Copenhagen, Copenhagen, Denmark

**Keywords:** heart transplantation, labor market participation, socioeconomic position, multimorbidity, public transfer payments

## Abstract

Most studies on vocational rehabilitation after heart transplantation (HTX) are based on self-reported data. Danish registries include weekly longitudinal information on all public transfer payments. We intended to describe 20-year trends in employment status for the Danish heart-transplant recipients, and examine the influence of multimorbidity and socioeconomic position (SEP). Linking registry and Scandiatransplant data (1994–2018), we conducted a study in recipients of working age (19–63 years). The cohort contained 492 recipients (79% males) and the median (IQR) age was 52 years (43–57 years). Five years after HTX, 30% of the survived recipients participated on the labor market; 9% were in a flexible job with reduced health-related working capacity. Moreover, 60% were retired and 10% eligible for labor market participation were unemployed. Recipients with multimorbidity had a higher age and a lower prevalence of employment. Five years after HTX, characteristics of recipients with labor market participation were: living alone (27%) versus cohabitation (73%); low (36%) versus medium-high (64%) educational level; low (13%) or medium-high (87%) income group. Heart-transplant recipients with multimorbidity have a higher age and a lower prevalence of employment. Socioeconomically disadvantaged recipients had a lower prevalence of labor market participation, despite being younger compared with the socioeconomically advantaged.

## Graphical Abstract

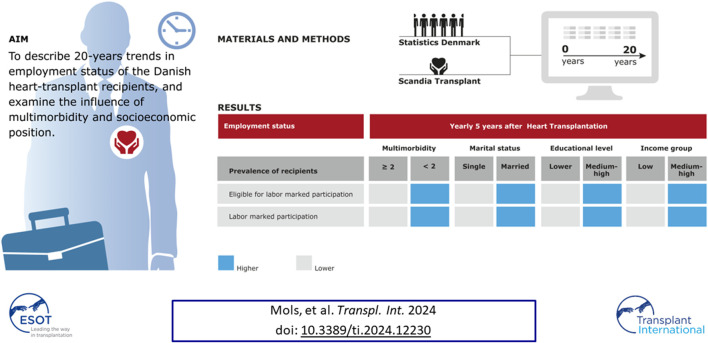

## Introduction

Heart transplantation (HTX) has become the definitive treatment influencing survival rates and quality of life in patients with end-stage heart failure (HF) [1, 2]. Worldwide, the median survival of adult heart-transplant recipients is 12 to 13 years and the 1-year survival rate is 91% [3]. During the last 30 years, advances in surgical techniques, perioperative management, and immunosuppressive therapies directed at acute graft rejection have improved life expectancy in heart-transplant recipients [3–5]. The improved prognosis has facilitated more attention to employment status post-HTX [2, 6, 7]. Employment is known to be essential for social identity, self-esteem, self-confidence, as well as mental and physical health in patients with HF [8, 9]. Moreover, labor market contribution carries economic advantages for both patients and society [8, 9].

Previous studies have reported a wide variation in the prevalence of return to work (31%–71%) in heart-transplant survivors [10–13]. Yet, studies on employment status after HTX have generally been based on questionnaires or other self-reported data. In Denmark, it is possible to make robust descriptions of labor market dynamics following HTX due to well-established and highly complete population-based registers and weekly longitudinal information on all public transfer payments from Danish authorities [14, 15]. A nationwide study in Denmark of patients with HF suggested that multimorbidity is associated with reduced chance of return to work within 1-year after first-time hospitalization [8]. Moreover, socioeconomic deprivation is known to be more likely in individuals with multimorbidity [16–19]. Improved insights into the influence of multimorbidity and socioeconomic position (SEP) on labor market participation in heart-transplant recipients may contribute to strategies to strengthen social recovery. Therefore, in this setting, we sought to describe 20-year trends in employment status for the Danish heart-transplant recipients, and examine the influence of multimorbidity and SEP.

## Materials and Methods

### Setting and Data Sources

Denmark has a primarily tax-financed universal healthcare system with free access to healthcare for all residents regardless of employment status [15]. Moreover, public welfare benefits and other social services are obtainable for all residents. Denmark has a rich infrastructure of nationwide administrative and clinical registers of high quality [15]. All residents are assigned a permanent and unique 10-digit identifier that allows for both accurate linkage at individual level and complete long-term follow-up [20].

In the present study, we used data from the following nationwide registers:

⁃ Scandiatransplant Database (STD), which collects clinical data on all Danish heart-transplant recipients since 1983 [21]. It is mandatory for hospitals to report to this database.
⁃ The Danish Rational Economic Agent Model (DREAM) Database, integrated data on social service payment transfers. DREAM contains weekly information on residents receiving any kind of public welfare payments since 1991 [14].
⁃ The Danish National Patient Registry (DNPR) [22] containing information on discharges from somatic hospitals since 1977 and from outpatient and emergency room visits since 1995. Records include dates of admission and discharge, discharge diagnosis according the International Classification of Diseases (ICD-8 and since 1994 ICD-10 codes), along with codes for diagnostic and surgical procedures.
⁃ The Psychiatric Central Research Register (PCRR) [23] containing information on all inpatient psychiatric diagnoses since 1969 and outpatient diagnoses since 1995.
⁃ The Danish National Prescription Registry (NPR) [24], established in 1995 and containing data on all prescriptions reimbursed at Danish community pharmacies. Prescribed pharmacotherapies are coded according to the Anatomical Therapeutic Chemical [ATC] Classification system.
⁃ Statistics Denmark’s [25] education and income registers, which contain information on highest level of completed education and income.
⁃ The Danish Civil Registrations System (CRS) [20] contains data on vital status, date of birth, gender as well as cohabitation and marital status with daily update.

### Study Cohort and Definitions

Denmark has two transplant centers—at the University Hospital of Copenhagen and at Aarhus University Hospital. We identified a national cohort of Danish recipients who underwent first-time HTX between 1 January 1994, and 31 December 2018 by the STD. The index date was defined as the date of the first HTX in the STD. We retrieved data on age, gender, and civil status from the CRS [20]. We restricted the cohort to recipients in working age (19–63 years) at index date and alive on discharge date. This upper age limit was chosen to identify those with a labor market expectation of at least 1-year before retirement, typically at the age of 65 years. Recipients who were not recorded in the DREAM register (*n* = 9) were excluded. The number of heart-transplant recipients alive at end of follow-up was counted. In accordance with a national Danish HF study [8], age at index date was divided into three categories (19–40, 41–50, and +51 years). Time since HTX was defined as 0–1, >1–10, and >10 years.

We identified non-psychiatric and psychiatric conditions by ICD codes registered in the DNPR [15] 10 years before index date (primary and secondary diagnoses) (Supplementary Table S1). Treatment with pharmacotherapies was defined as ≥1 redeemed prescription 6 months before index date according to the NPR [15]. The number of cardiovascular pharmacotherapies was summarized, and polypharmacy was defined as redeeming at least one prescription for ≥5 different cardiovascular agents [15] (Supplementary Table S2).

Consistent with prior definitions of multimorbidity in Danish studies [26, 27], we calculated the number of non-psychiatric and psychiatric conditions 10 years before index date based on data obtained from the DNPR [15, 22] and the PCRR [15, 23]. The used algorithm estimated multimorbidity as the co-occurrence of two or more chronic conditions included in 11 comprehensive chronic disease groups. We summarized the number of multimorbidity based on the count of chronic disease groups, excluding all cardiovascular diseases (Supplementary Table S1).

We retrieved individual-level SEP data from the CRS and Statistics Denmark the year before index date [20, 25]. The SEP compiled information on cohabitation status, marital status, educational level, and personal income group. Cohabitation status was defined as living alone or cohabitation (living with other individuals) and marital status as married (registered partnership) or single (including never or not yet married, divorced, or widowed). Based on international standard classification, highest educational level was divided into three groups: primary and lower education (low); upper secondary education and academy profession (medium); bachelor and above (high). To account for inflation and salary changes over time, we determined personal income (pre-tax total) based on the annual percentiles in the Danish population. We classified personal income into percentiles and used the 25th percentile as cut-off point for low (≤25th percentile) and medium-high (>25th percentile) income (Supplementary Table S3). Average 5-year household income was not included since data on household income statistics was only available after 2004 in Danish registers.

### Labor Market Participation

We complied information from the DREAM register on labor market participation [14]. For each week starting 1 year before the index date (week −52) and continuing for up to 5 years, we grouped patients into six categories of employment status according to the type of social transfer benefits received: regular employment (i.e., employed or receiving parental leave payments, benefits due to sick child, or vacation payments); flexible job (a job for those with reduced health-related working capacity); health-related work absenteeism (i.e., sick leave, unemployed awaiting flexible job, rehabilitation, or work ability clarification); unemployment, not health related (i.e., unemployment benefit or social assistance, not health related); retirement (early retirement pensions, post-employment retirement, retirement); censoring or death. The lengths of work disability periods financed by the employer (not recorded in DREAM) have varied between 14 and 30 days during the 20-year period-analyzed (Supplementary Table S4). Baseline employment status was identified by the week before the week of HTX (week −1).

### Statistical Analysis

Continuous baseline characteristics were presented as median values with 25th–75th interquartile range (IQR) because the observed skewedness in the data. Categorical variables were presented as numbers and percentages. Recipients were followed until 1 January 2019, death, or emigration, whichever came first.

To describe the weekly changes in employment status 1 year before (week −52) and 2 years after (week 104) index date, we graphically illustrated prevalence of each of the following categories for each week: regular employment; flexible job; health-related work absenteeism; unemployment, not health related; retirement; censoring or death (Table 1) (Supplementary Table S4). We have followed the Statistics Denmark’s rules on sensitive and personal data and therefore we do not report aggregated results based on less than five observations or numbers below five. Thus, the “education” category was merged together with the category “unemployed, not health related.” Moreover, we hided the categories of “censored” or “death” until there were at least five patients in each of them.

**TABLE 1:** Classification of labor marked participation.

Categorization	Weekly employment status	Sankey diagram	Employment status by multimorbidity and SEP	
Education	Unemployed, not health related	-	-	-
Regular employment	Regular employment	Regular employment	Labor marked participation	Eligible for labor marked participation
Flexible job	Flexible job	Reduced workability		
Unemployed, not health related	Unemployed, not health related	-	-	
Health-related work absenteeism	Health-related work absenteeism	Reduced workability	-	
Retirement	Retirement	Retirement	-		-
Emigrated	Censored or Death	-	-		-
End of study					

SEP, socioeconomic position.

Sankey flow illustration is a technique for data visualizations that emphasizes movement or flow from one state to another [28]. We used the Sankey graphical illustrations to display the movements between heart-transplant recipients in regular employment, with reduced work ability, and retired 1 year before (week −52) to 1 year (week 52) after index date. We defined reduced work ability by recipients in flexible job or at health-related work absenteeism because recipients in these social payment groups are unable or have limited ability to work due to illness. However, they do not receive permanent retirement benefits and thus have the possibility for increased vocational rehabilitation (Table 1) (Supplementary Table S4).

To examine the influence of multimorbidity and SEP on employment status and to identify the most vulnerable heart-transplant recipients presumably susceptible to reduced labor market participation, we stratified the analyses by all the variables of multimorbidity and SEP: degree of multimorbidity (0-1 versus 2+); cohabitation status (living alone versus cohabitation); marital status (single versus married); educational level (low versus medium-high [medium or high]); income group (low versus medium-high). To specifically describe the pattern of labor market participation 1 year before (year-1) HTX and annually within 5 years of follow-up (year 1, year 2, year 3, year 4, and year 5), we graphically displayed the prevalence of heart-transplant recipients by labor market participation, within the group of recipients eligible for labor market participation, overall as well as by the stratifying variables of multimorbidity and SEP. Heart-transplant recipients defined as ineligible for labor market participation included: education; retirement; emigrated; end of study; deaths. Heart-transplant recipients identified with labor market participation included individuals with regular employment or flexible jobs (Table 1) (Supplementary Table S4).

Analyses were conducted using the SAS Statistical Software version 9.4 (SAS Institute, Cary, NC) and R version 4.1.0 (2021-05-18).

## Results

A total of 649 heart-transplant recipients were identified during the study period. Of these, 492 were of working age (19–63 years) at the time of surgery, registered in the DREAM database and alive at discharge. The cohort contained 390 males (79%) and the median (IQR) age was 52 years (43–57 years). The three most prevalent non-psychiatric conditions before index-date in the HTX cohort were HF (87%) followed by cardiomyopathy (67%) and cardiac arrhythmias (48%). Psychiatric conditions were observed in less than 1% of recipients. The median (IQR) number of both non-psychiatric (excluding cardiovascular diseases) and psychiatric conditions was 1 (1-2) (Table 2).

**TABLE 2:** Baseline characteristics of heart-transplant recipients.

	Total
	*N* = 492
Gender	
Male	390 (79)
Female	102 (21)
Age	
Median (IQR)	52 (43–57)
Age groups	
19–40	114 (23)
41–50	342 (70)
+51	36 (7)
Follow-up time in years	
0–1	33 (7)
1–5	115 (23)
5–10	122 (25)
10+	222 (45)
Alive at end of follow-up	306 (62)
Non-psychiatric conditions (10 years prior to the index date)	
Cardiovascular disease	
Myocardial infarction	182 (37)
Angina Pectoris	216 (45)
Heart failure	429 (87)
Heart valve diseases	58 (12)
Cardiac arrhythmias	237 (48)
Congenital heart disease	34 (7)
Cardiomyopathy	328 (67)
Cardiac inflammation	48 (10)
Aortic disease	NA
Peripheral arterial disease	10 (2)
Cerebrovascular disease	42 (9)
Cardiogenic shock and pulmonary edema	48 (10)
Hypertension	62 (13)
Diabetes	64 (13)
Chronic obstructive pulmonary disease	49 (10)
Cancer	19 (4)
Chronic neurological disease	8 (2)
Chronic arthritis	NA
Chronic bowel disease	7 (1)
Chronic liver disease	11 (2)
Chronic kidney disease	24 (5)
Psychiatric conditions (10 years prior to the index date)	NA
Multimorbidity (10 years prior to the index date)	
Number of chronic diseases, median (IQR)	1 (1–2)
Cardiovascular polypharmacy (6 months prior to the index date) ^a^	
Prescribed medications ≥5 Cohabitation status	276 (56)
Living alone	141 (29)
Cohabitation	351 (71)
Marital status	
Single	191 (39)
Married	301 (61)
Highest obtained educational degree	
Low (primary and lower secondary education)	151 (31)
Medium (upper secondary education and academy profession degree)	234 (48)
High (bachelor and above)	95 (19)
Missing	12 (2)
Personal income group	
Low income (≤25th percentile)	57 (12)
Medium-high income (>25th percentile)	435 (88)
Employment status	
Regular employment	77 (16)
Flexible job	28 (6)
Unemployment, not health related	17 (3)
Health-related work absenteeism	152 (31)
Retirement	218 (44)

Values are *n* (%).

NA, not available due to data protection.

^a^ Data available since 1995 in the Danish National Prescription Registry.

**FIGURE 1:**
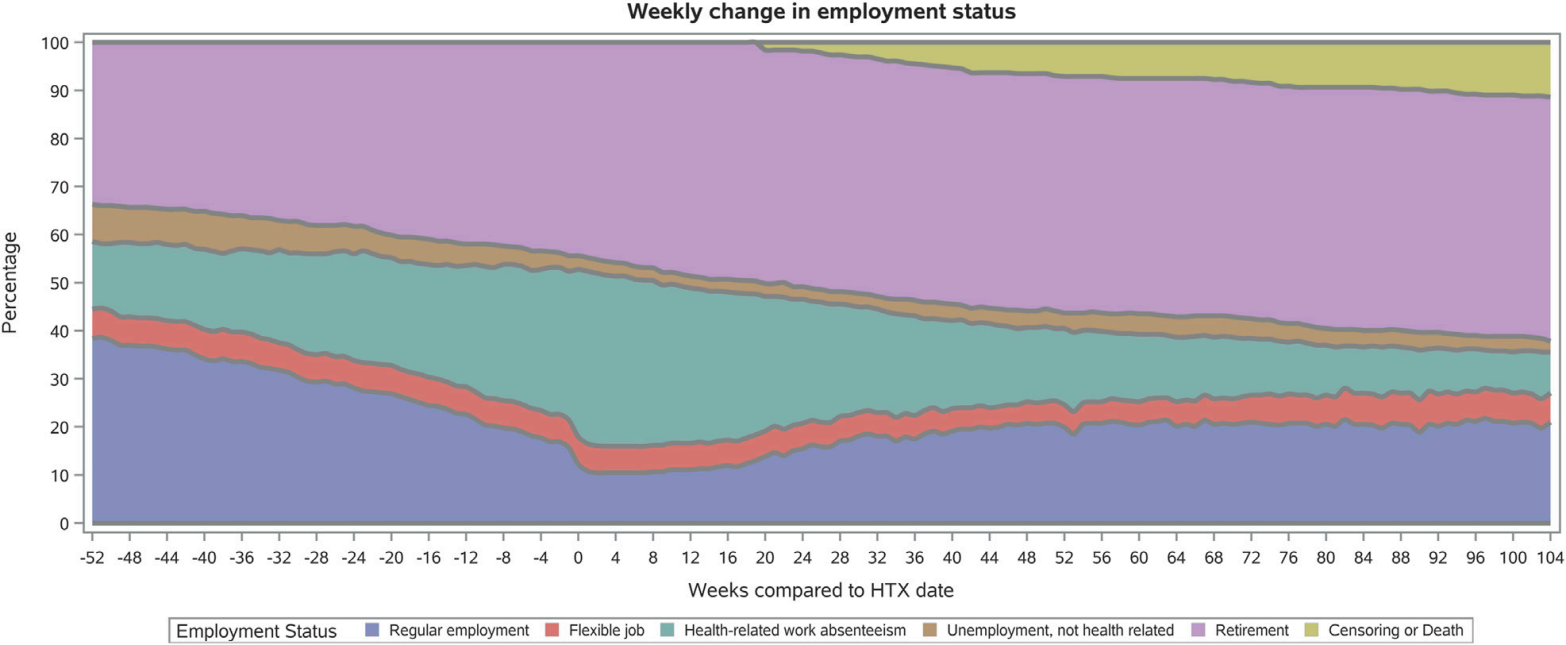
Weekly employment status in heart-transplant recipients from 1 year before to 1 year after surgery. HTX, heart transplantation.

Figure 1 displays the dynamic patterns in weekly changes in employment 1 year before and up to 2 years after HTX. The prevalence of regular employment 1 year before index date was 38%, decreasing to 12% at surgery, and increasing to 21% 2 years after index date. Recipients in a flexible job showed a stable pattern with approximately 6% having reduced health-related working capacity during the study period. The prevalence of heart-transplant recipients with health-related work absenteeism was highest at index date (35%), whereas the prevalence was 9% 2 years after surgery. Eight percent of the recipients were unemployed, not health related, 1 year before HTX versus 2% after 2 years. The prevalence of recipients on retirement increased the year up to HTX, while the prevalence was steady and approximately 50% 2 years after surgery. Four weeks before HTX, the prevalences were approximately: regular employment (18%); flexible job (6%); health-related work absenteeism (29%); unemployment, not health related (4%); retirement (43%).

**FIGURE 2:**
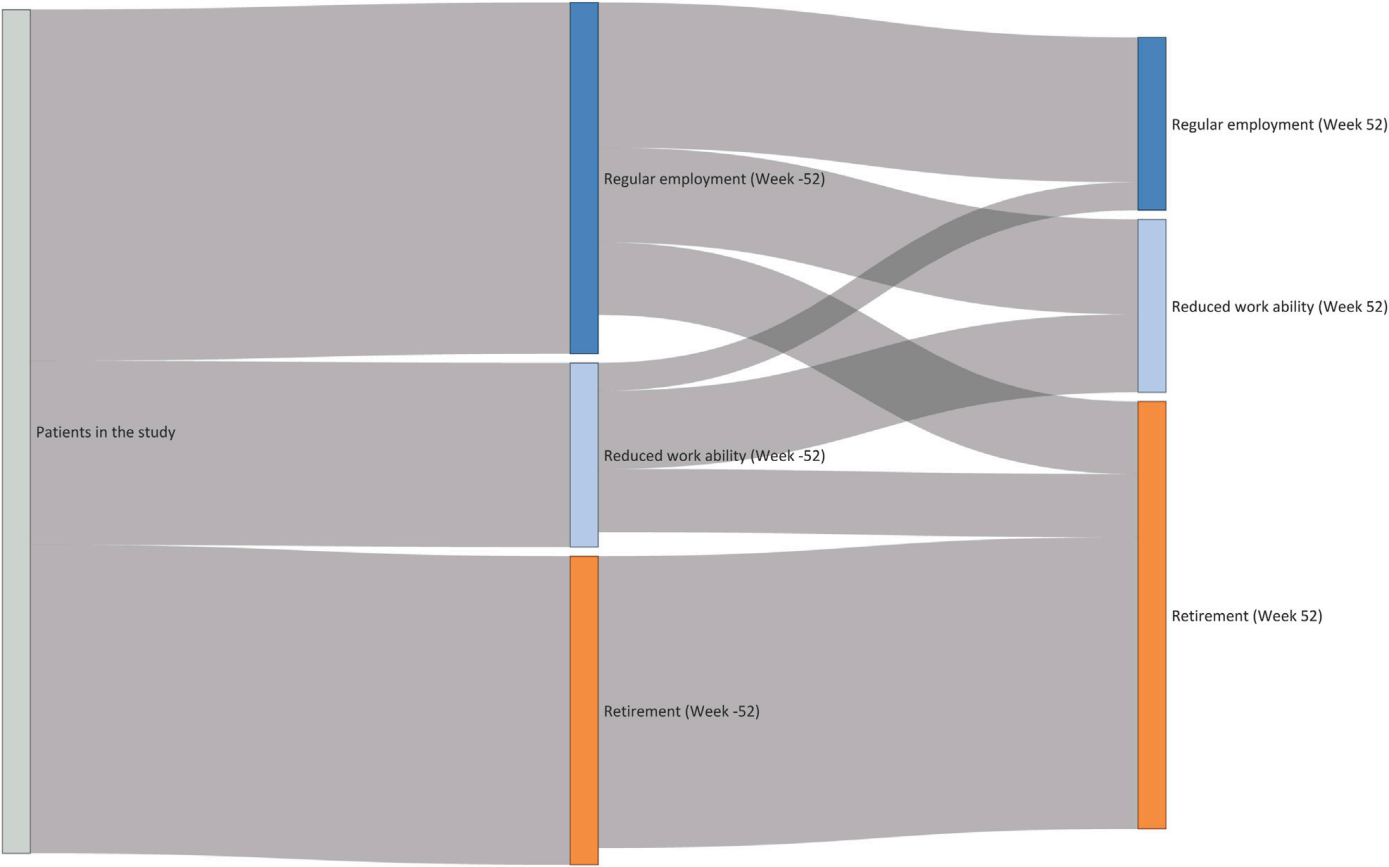
Movements from 1 year before to 1 year after surgery within the groups of heart-transplant recipients in regular employment, with reduced work ability, or on retirement.

Through the Sankey flow diagram in Figure 2, movements from 1 year before to 1 year after index date were observed between the groups of heart-transplant recipients in regular employment, with reduced work ability, or on retirement. Twenty-one percent of recipients with regular employment as well as 34% with reduced work ability moved to the retirement group. Within the group of recipients with regular employment, 27% moved to the group with reduced workability from 1 year before to 1 year after HTX. The movement from reduced work ability to regular employment was observed in 15% of the heart-transplant recipients.

**FIGURE 3:**
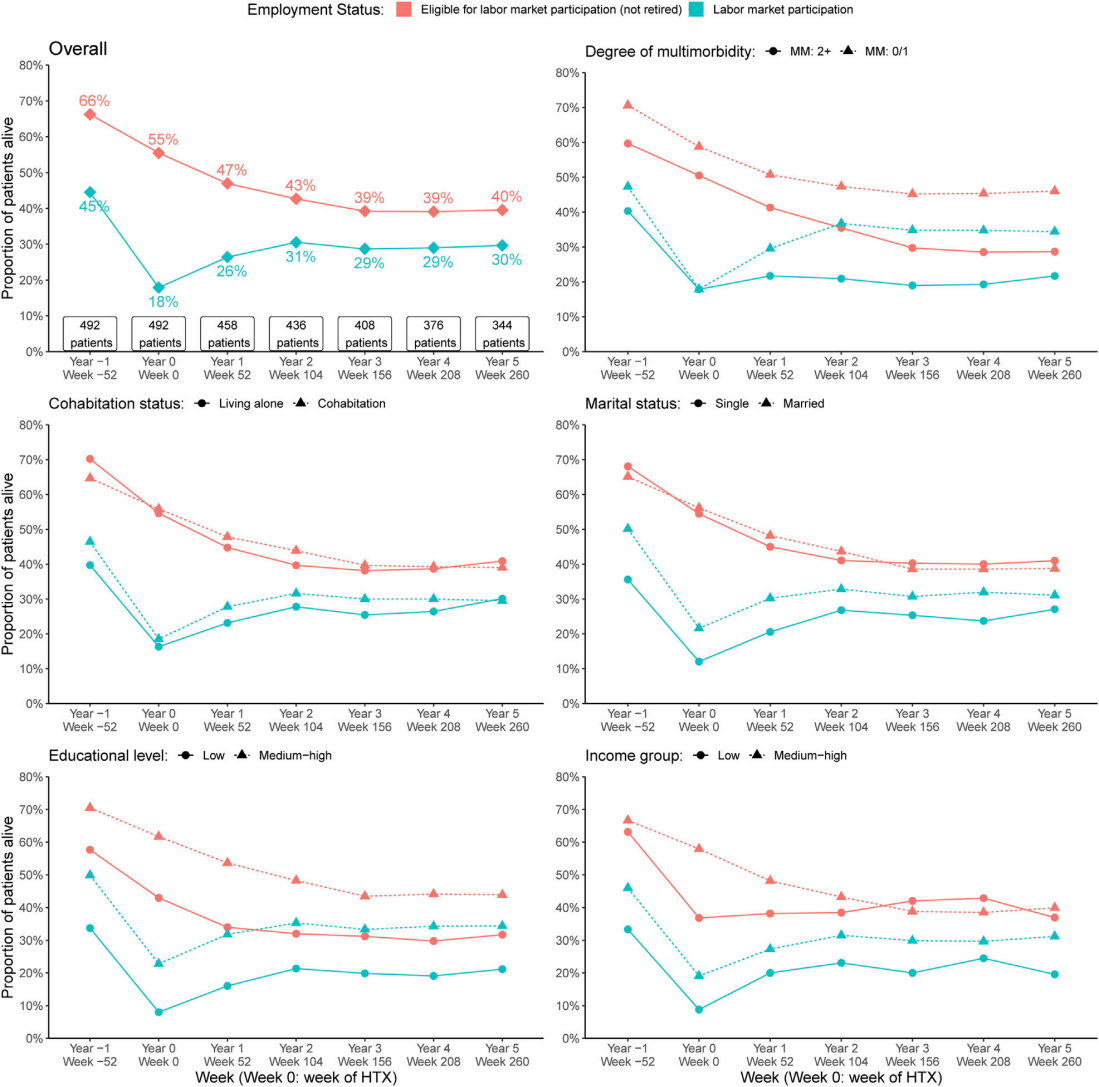
Prevalence estimates of labor market participation within the group of recipients eligible for labor market participation at 1 year before HTX to 5 years after. The prevalences are depicted overall and by the stratifying variables of multimorbidity, cohabitation status, marital status, educational level, and income group. HTX, heart transplantation; MM, Multimorbidity.

**FIGURE 4:**
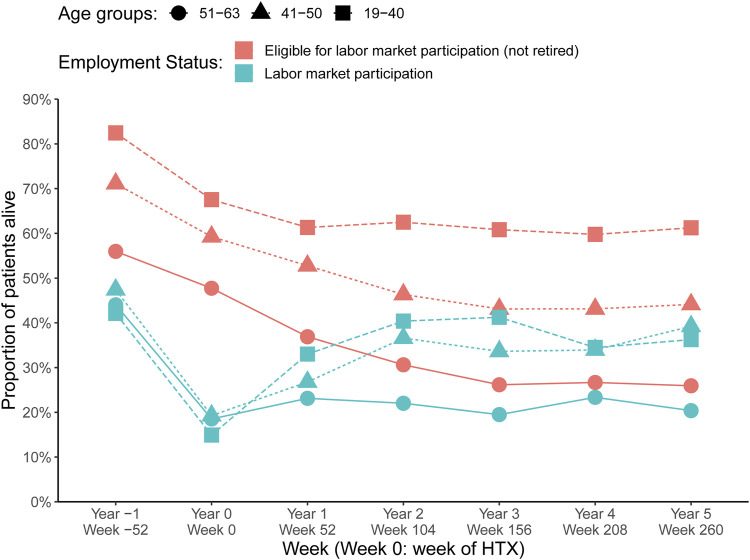
Labor market participation by age groups HTX, heart transplantation.

Prevalence estimates of labor market participation within the group of recipients eligible for labor market participation at 1 year before HTX to 5 years after as well as prevalence by the stratified variables of multimorbidity and SEP are depicted in Figure 3. One year after HTX, 26% of the recipients participated in the labor market, increasing to 30% after 5 years (9% in a flexible job; 21% in a regular job). In the time between 2–5 years after index date, approximately 10% eligible for labor market participation did not participate on the labor market. Those recipients within the lowest age interval (19–40 years) compared to the highest age interval (51–63 years), presented higher prevalence with labor market participation (Figure 4). In recipients with at least two chronic diseases, the prevalence of both recipients eligible for and with labor market participation was lower compared with recipients with no more than one chronic disease (Figure 3). Of notice, we found no indication of differences in age between recipients with 0-1 versus 2+ chronic diseases (Supplementary Figure S1). We observed a socioeconomic influence in labor market participation. In heart-transplant recipients living alone, being single, with low educational level, or in the lowest income group, the prevalence of labor marked participation was lower during the study period, though less pronounced in recipients living alone (Figure 3). Except for educational level, the most socioeconomically disadvantaged heart-transplant recipients were younger (Supplementary Figure S1). Five years after HTX, characteristics of recipients with labor market participation were: living alone (27%) versus cohabitation (73%); single (35%) versus married (65%); low (36%) or medium-high (64%) educational level; low (13%) or medium-high (87%) income group.

## Discussion

Our nationwide study in heart-transplant recipients is the first to describe long-term employment dynamics with a high weekly accuracy. We had several novel findings: i) 5 years after HTX, 30% of the recipients participated on the labor market, 9% of those were in a flexible job; ii): sixty percent of survived recipients were retired as well as 10% of recipients eligible for labor market participation was unemployed 5 years after HTX; iii) recipients with multimorbidity had a higher age and a lower prevalence of employment; iv) socioeconomically disadvantaged heart-transplant recipients had a lower prevalence of labor market participation, despite being younger than the socioeconomically advantaged.

Previous studies have reported return to work following HTX [10–12, 29]. However, these studies did not describe detailed labor market participation by public transfer payments from authorities. White-Williams et al. performed [13] a questionnaire survey study in 237 heart-transplant recipients from the University Medical Centers in the United States (US). Pre-transplant (listed for HTX date), 17% of recipients were active on the labor market, increasing to 26% 1 year after surgery. Only, 45% of the recipients working 1-year post-transplant had also been working pre-transplant [13]. Kristen et al. (2009) [10] forwarded a questionnaire to 150 heart-transplant recipients 8.5 ± 0.4 years after surgery at a German university hospital. Thirty-six percent of recipients were of working age (<65 years) and employed after 12.6 ± 1.9 months [10]. A single-center study (2019) [11] included survived recipients attending a Scottish heart-transplant clinic (*n* = 154). One year before HTX, 61% (*n* = 47) of recipients were working. Of those, 83% (*n* = 39) returned to work and 21% had reduced working hours [11]. In the newly presented UNOS (United Network for Organ Sharing) database study, they addressed the prevalence of employment following HTX in the US [12]. The study included 10,132 recipients who survived at least 1-year (75% were males). Median (IQR) age in the non-working group was 51 years (42–56) versus 49 years (39–55 years) in the working group. Twenty-two percent of survived recipients were employed 1 year after surgery and employment prevalence of 2-year survivors increased to 32.9%. Within the group of recipients with employment 1-year after HTX, 62.8% were not working at listing or surgery date. Moreover, 16.1% who were not working at listing or surgery date obtained a working position afterwards [12]. Likewise, a newly published systematic review [30] on employment following thoracic transplantation reported that employment ranged from 19.7% to 69.4% for heart-transplant recipients [30]. The findings from the review and individual studies varied considerably. Discrepancies between these results may be explained by a variety of reasons, including inclusion criteria (e.g., working age interval; labor market participation status at index data), measures of labor market participation (e.g., questionnaire-based data; other self-reported data; question-asking technique at clinical visits), and national government set-up (e.g., universal public welfare benefits; other social services or more selective welfare stats).

Our register-based approach allowed us to illustrate the movements in labor market participation dynamically 1 year before and up to 5 years after HTX. Our findings confirm the results based on the UNOS database with higher accuracy and prolonged long-term follow-up. Thus, approximately 30% of the survived heart-transplant recipients were employed long-term after surgery (21% in a regular job as well as 9% in flexible job). Additionally, we observed that only 5% of recipients moved from reduced workability to regular employment within the first year after HTX and the prevalence of retired recipients increased from 35% to 60%. These observations support the idea that with vocational rehabilitation, many heart-transplant recipients are able to participate on the labor market [2]. However, as shown in our data, 10%–12% of the recipients eligible for work are unemployed in the period 2–5 years after HTX, which could reflect missed facilitation of social recovery.

Several factors have been identified to influence labor market participation. The UNOS database study [12] found that independent predictors of obtaining employment after HTX were age, gender, employment status at time of listing or transplant, education, insurance status, ethnicity, and postoperative complications. However, the most influential predictor was reported to be the preoperative employment status [12]. Similarly, the review regarding thoracic transplantation reported that the most positively associated factors with employment post-surgery were younger age, higher educational level, and pre-surgery employment, whereas the most negatively associated were longer duration of unemployment before transplant and Medicaid coverage [30]. Unlike these studies, we described the impact of multimorbidity and SEP on labor market participation. Our study showed that multimorbidity reduced both the prevalence of recipients eligible for labor market participation and the number of recipients with labor market participation during a period of 5 years after HTX. The Danish nationwide register-based cohort study [8] among first-time hospitalized patients with HF patients (*n* = 21,455) reported that comorbidities such as stroke (OR 0.55; 95% CI 0.45–0.69), chronic kidney disease (OR 0.46; 95% CI 0.36–0.59), and diabetes mellitus (OR 0.76; 95% CI 0.68–0.85) were all associated with a reduced likelihood of return to work [8]. Another Danish study [31] including 5,365 patients hospitalized with cardiac disease (mean [SD] age 50 years [11]; 70% males) reported that poor self-reported physical and mental health and high symptom burden were associated with detachment from the workforce within 1-year after discharge [31]. Along with our results, these studies support that heart-transplant recipients with the ability to work may have a better physical function, less limitation in their activities, lower age, and male gender.

Our results could indicate socioeconomic differences in workability following HTX. In accordance with the previous UNOS study [12], we found that in recipients with a lower level of education or income, the prevalence of both eligibility and participation on the labor market was lower. A possible explanation could be that individuals employed and having a low SEP are known to be more predisposed to unfavorable psychosocial working conditions and more likely face mental and social problems outside the workplace compared with those employed with a high SEP [32]. Thus, they might experience additional barriers to labor market participation. Accordingly, employment-related barriers for workability, such as less physically demanding jobs in individuals with a high educational level, is reported to facilitate more successful reintegration into the labor market [9, 12, 30, 32]. The slightly lower prevalence of both eligibility and labor market participation in heart-transplant recipients living alone or unmarried reflects that social support from a partner might result in improved self-management and physical recovery in heart-transplant recipients. This was supported by a recent Danish study [27] in heart-transplant recipients (*n* = 649; 78% males, 59% in age interval 41–60 years) suggesting a higher risk of first-time MACE (Major Adverse Cardiovascular Event) within 1–10 years after surgery (HR 1.46, 95% CI 0.98–2.17). In addition, the study illustrated that low educational level (adjusted HR 1.66, 95% CI 1.14–2.43) and low income (adjusted HR 1.81, 95% CI 1.02–3.22) were associated with a first-time MACE [27]. In agreement with our findings, these observations indicate a higher illness burden in socioeconomically disadvantaged heart-transplant recipients, thus leading to reduced workability.

In agreement with previous studies, our observations underline the call for more special attention to address employment status in the target group of socioeconomically disadvantaged heart-transplant recipients with multimorbidity. However, more research is needed into strategies to support workforce reintegration continuously and long-term after HTX. Systematic screening of heart-transplant recipients at follow-up visits for self-reported psychical, social, and mental factors associated with reduced labor market participation may be beneficial.

The major strengths is the use of nationwide administrative and clinical registries with complete and unselected information. These registries made it possible to present weekly, detailed and updated data on labor market participation with complete and long-term follow-up during 1994–2018. This strengths is essential compared to the UNOS-STAR database study (part of the International Society for Heart & Lung Transplantation), where employment status is documented by systematic questions at the time of post-transplant follow-up visits.

Our study also has some limitations. Given the reliance on the DREAM register, visibility into health-related disability payments was reduced to governmental expenses with no information pertaining to employer disability. Moreover, the first 2 weeks of any social transfer are not recorded in the DREAM register as the employer covers costs. This limits a complete understanding of labor market dynamics, but likely only for those who are disabled for a very short period, as those disabled for longer periods would typically receive governmental benefits. Consequently, this is unlikely to impact the study findings. Another limitation is that this study is observational and has a small sample size. We have a descriptive study design and as such, we could not assess any confounding effects. Thus, descriptions and illustrations may not be interpreted as associations or causations. Finally, the transferability of our findings may only be relevant to other European countries with a social structure and a public welfare system similar to Denmark.

Five years after HTX, 30% of recipients participated on the labor market, whereas 10% of recipients eligible for labor market participation were unemployed. Recipients with multimorbidity have a higher age and a lower prevalence of employment. Socioeconomically disadvantaged heart-transplant recipients displayed a lower prevalence of labor market participation, despite being younger compared with the socioeconomically advantaged.

## Data Availability

Study data, statistical plan, and log-files can be made available through proposal to the Project Database (ID: 707738) at Statistics Denmark. Requests to access the datasets should be directed to https://www.dst.dk/en/TilSalg/Forskningsservice.

## Abbreviations

ATC, Anatomical Therapeutic Chemical Classification system.; CRS, The Danish Civil Registrations System; DNPR, The Danish National Patient Registry; NPR, The Danish National Prescription Registry; DREAM, The Danish Rational Economic Agent Model Database; HF, Heart Failure; HTX, Heart transplantation; ICD, International Classification of Diseases; IQR, Interquartile Range; PCRR, The Psychiatric Central Research Register; SEP, Socioeconomic Position; STD, Scandiatransplant Database.
