# Supplementary material for: Employment Status Following Heart Transplantation: Data From the Danish Nationwide Social Service Payment Register During 20 years
Source: Transpl Int. 2024 Apr 17;37:12230. doi: 10.3389/ti.2024.12230 (PMC11061463; doi:10.3389/ti.2024.12230)

Online SUPPORTING MATERIAL

Employment status following heart transplantation: Data from the Danish nationwide social service payment register during 20 years

Contents

Table S1 International Classification of Diseases codes of algorithm to measure multimorbidity

Table S2 Anatomical Therapeutic Chemical Classification Codes of cardiovascular medical treatment

Table S3 Classification of individual-level socioeconomic position

Table S4 Classification of labor marked participation

Figure S1 Median (interquartile range) age by categories of multimorbidity and socioeconomic position

Table S1 International Classification of Diseases codes of algorithm to measure multimorbidity [1-4]

Included CDGs (grey) and included diseases (white)	ICD-8	ICD-10
**Cardiovascular disease [5,6]**		
Myocardial infarction	410	I21
Angina Pectoris		
Unstable	411	I200
Stable	413	I20 (without I200), I251, I259
Heart Failure	42709, 42710, 42711, 42719, 42899,78249	I500, I501, I502, I503, I508, I509, I110, I130, I132, I420, I426, I427, I428, I429
Heart Vale disease		
Mitralvalve-insufficiency and stenosis	394	I05, I34, I390, I511A
Aorticvalve-insufficiency and stenosis	395	I06, I35, I391
Cardiac arrhythmia		
Atrial fibrillation or flutter	42793, 42794	I48
Bradycardia (sinus node dysfunction and AV-block)	42720, 42721, 42722, 42723	I440, I441, I442, I443, use 145 (*I455A, I455B, I455C, I455G)*
Ventricular tachycardia/fibrillation	42797, 42791	I470, I472, I490
Cardiac inflammation		
Endocarditis	421	I33, I38, I398
Myocarditis	422	I40, I41, I090, I514
Pericarditis	39109, 393, 420, 423	I30-132
Aortic disease		
Aortadissection	44109	I710
Aneurisme/dilatation	44110, 44111, 44119, 44120, 44121, 44129, 44199	I711-I716, I718-I719
Peripheral arterial disease	44389-44399	I739A
Cerebrovascular disease	430-438	I60-I69, G45, G46
Cardiogenic shock and pulmonary edema	427.10, 427.11	J81, I501B, R570
Congenital heart disease	746.00–747.49, 759.00, 759.01, 759.09	Q200-Q269, Q893
Cardiomyopathy	425	I42-I43 (excluding I42.6)
**Hypertension**		

Hypertension	400-404	I10-I15
**Diabetes**		
Diabetes with end organ damage type1 type2	249.00, 249.06, 249.07, 249.09, 250.00, 250.06, 250.07, 250.09	E10-E14. O24 (except O24.4), G63.2, H36.0, N08.3
**Chronic obstructive pulmonary disease** [5,6]		
COPD	490-493; 515-518	J40–J47; J60–J67; J68.4; J70.1; J70.3; J84.1; J92.0; J96.1; J98.2; J98.3
**Cancer** [5,6]		
Cancer	140–209	C00-C97
**Chronic neurological disease** [6]		
Epilepsy	345	G40 (ex. G40.4), G41
Parkinson's disease	342	G20-G22
Multiple sclerosis	340	G35
**Chronic arthritis** [6]		
Rheumatoid arthritis/connective tissue disease	696.09, 712, 715	L40.5, M05-M07
**Inflammatory bowel disease/Chronic bowel disease** [6]		
Colitis ulcerosa and Mb. Crohn	563	K50-K51
**Chronic liver disease** [6]		
Chronic viral hepatitis,<br>Chronic liver disease	571-573	B18<br>K70-K76
**Chronic kidney disease** [6]		
Chronic pyelonephritis/Interstitial nephritis	590.09, 593.20	N11, N14, N15, N16
Other and unknown chronic renal disease (none of those above):	792, 584	N18-N19, N26, N27, N07, N08 (without N08.3)
**Chronic mental disease** [5,6]		
Schizophrenia	295.x9, 296.89,	F20 –F29
And related disorders/psychotic disorders	297.x9, 298.29-298.99, 299.04, 299.05, 299.09, 301.83	
Affective disorders incl. depression	296.x9 (excluding 296.89), 298.09, 298.19, 300.49, 301.19	F30-39

Dementia		
Alzheimer's disease	290.10	F00 (includes F00.0x, F00.1x, F00.2x, and F00.9x); G30 (includes G30, G30.0, 30.1, 30.8, 30.9)
Vascular dementia	293.09, 293.19	F01 (includes F01.0x, F01.1x, F01.2x, F01.3x, F01.8x, & F01.9x)
Other dementia	094.19 and 292.09; 290.09, 290.11, 290.18, 290.19, 292.09	F02; F03; F05.1; F1x.73 (F10.73 through F19.73); G23.1; G31.0A*, G31.0B*, G31.1, G31.8B, G31.8E
Other exclusions: Mild cognitive impairment (MCI) and amnestic syndromes. Applies when identifying cases and controls free from dementia. Not used to identify incident dementia	291.19	F04, F04.9, F05.1, F06.7 and F06.7x; F1x.6 (F10.6, F18.6, F19.6)
Neurotic, stress-related and somatoform disorder	300.x9 (excluding 300.49), 305.x9, 305.68, 307.99	F40-F48
Eating disorder	305.60, 306.50, 306.58, 306.59	F50

CDG = Comprehensive groups of Chronic Diseases; ICD = International Classification of Disease.

1. Schmidt M, Schmidt SA, Sandegaard JL, Ehrenstein V, Pedersen L, Sorensen HT. The Danish National Patient Registry: a review of content, data quality, and research potential. *Clinical epidemiology.* 2015;7:449-490.
2. Sundbøll J, Adelborg K, Munch T, et al. Positive predictive value of cardiovascular diagnoses in the Danish National Patient Registry: a validation study. *BMJ open.* 2016;6(11):e012832.
3. Adelborg K, Sundbøll J, Munch T, et al. Positive predictive value of cardiac examination, procedure and surgery codes in the Danish National Patient Registry: a population-based validation study. *BMJ open.* 2016;6(12):e012817.
4. Mors O, Perto GP, Mortensen PB. The Danish Psychiatric Central Research Register. *Scandinavian journal of public health.* 2011;39(7 Suppl):54-57.
5. Diederichs C, Berger K, Bartels DB. The measurement of multiple chronic diseases--a systematic review on existing multimorbidity indices. *The journals of gerontology Series A, Biological sciences and medical sciences.* 2011;66(3):301-311.
6. Barnett K, Mercer SW, Norbury M, Watt G, Wyke S, Guthrie B. Epidemiology of multimorbidity and implications for health care, research, and medical education: a cross-sectional study. *Lancet (London, England).* 2012;380(9836):37-43.

Table S2 Anatomical Therapeutic Chemical Classification Codes of cardiovascular medical treatment (1)

**Cardiovascular medical treatment**	**ATC Classification**
Cardiac therapy	C01
Anti-hypertensive	CO2
Diuretics	CO3
Peripheral vasodilators	CO4
Vasoprotective	CO5
Beta-blockers	CO7
Calcium antagonists	CO8
Renin-angiotensin system inhibitions	CO9
Lipid-modifying	CO10
Anticoagulants	B01A
Aspirin	B01AC06

ACT, Anatomical Therapeutic Chemical Classification.

1. Pottegard A, Schmidt SAJ, Wallach-Kildemoes H, Sorensen HT, Hallas J, Schmidt M. Data Resource Profile: The Danish National Prescription Registry. International journal of epidemiology 2017;46:798-798f.

Table S3 Classification of individual-level socioeconomic position [1-5]

**Individual-level socioeconomic position**		**Dichotomized**
Cohabitation status	Alone (single, divorced, widow, etc.)<br>Cohabitating (living with other individuals)	**Alone**<br>**Cohabitating**
Marital status	Married (registered partnership)<br>Single (including never or not yet married, divorced, or widowed)	**Married**<br>**Single**
Highest attained educational level	Low (no formal education; primary and lower secondary education)<br>Medium (upper secondary education and academy profession degree)<br>High (bachelor and above)<br>Missing	**Low education** (low degree)<br>**Medium-high education** (medium + high degree)<br><br>*(Missing not included)*
Personal income	Based on the annually reported (since 1995) nationwide personal (pre-tax total) income statistics, we selected the 25th, 50th and 75th percentiles and then used the 25th percentile as a cut-off value for a binary income categorization: lowest (≤ 25th percentile) and medium-high (> 25th percentile).<br><br>Data on personal (pre-tax total) income were obtained from the Income Statistics Register [5]<br>The annual personal (pre-tax-total) income 25th, 50th and 75th percentiles in the National Danish Population was available from 1995, and thus income was estimated by linear regression from 1993-1995. They are based on the numbers downloaded from the STATBAK.DK at January 13, 2021.<br><br>https://www.statbank.dk/statbank5a/SelectTable/Omrade0.asp?SubjectCode=04&ShowNews=OFF&PLanguage=1	**Low income** (≤25th percentile)<br>**Medium-high income** (>25th percentile)

1. Galobardes B, Shaw M, Lawlor DA, Lynch JW, Davey Smith G. Indicators of socioeconomic position (part 1). *Journal of epidemiology and community health.* 2006;60(1):7-12.
2. Schmidt M, Pedersen L, Sorensen HT. The Danish Civil Registration System as a tool in epidemiology. *European journal of epidemiology.* 2014;29(8):541-549.
3. Petersson F, Baadsgaard M, Thygesen LC. Danish registers on personal labour market affiliation. *Scandinavian journal of public health.* 2011;39(7 Suppl):95-98.
4. Jensen VM, Rasmussen AW. Danish Education Registers. *Scandinavian journal of public health.* 2011;39(7 Suppl):91-94.
5. Baadsgaard M, Quitzau J. Danish registers on personal income and transfer payments. *Scandinavian journal of public health.* 2011;39(7 Suppl):103-105.

Table S4 Classification of labor marked participation by DREAM[1] codes.

Entries	Description	Categorization	Weekly employment status	Sankey Diagram	Employment status by multimorbidity and SEP	
521	Adult trainee	Education	Unemployed, not health related<br>*(Data protection; ≤ 2%)*	-	-	-
651 652 661 662 794	State Education Fund grants					
413	Leave of absence due to education					
No entry	No transfer payment	Regular employment	Regular employment	Regular employment	Labor marked participation (*employed recipients*)	Eligible for labor marked participation *(not retired)*
795	Benefits due to sick child					
122 123	Vacation payment from employment					
412-413	Leave-of-absence schemes					
121	Vacation payment					
881	Maternity leave pay					
996	Retirement age, but without retirement payment					
761-762 769 771-774 779 782 796 895	Flexible job (a job for those with reduced health-related working capacity)	Flexible job	Flexible job	Reduced workability		
111 114	Unemployment benefit all week	Unemployed, not health related	Unemployed, not health related	-	-	
112 113 115	Unemployment benefit part time					
124-126	Vacation payment from unemployment					
130-139 141-142 152-153 730-739 741	Social assistance, not health related					
160 163-169	Ready for employment benefit					
710-719	Social benefit, immigrants					
704-709	Immigration benefit during job training					
140-149 151 414 700 703 720-729 732 742 751 752	Education assistance, not health related					
211-219 221 222 224 225 231 232 297-299 511 522 541 722 759	Unemployment benefit during special efforts e.g. job training or supervision.					
774 890 891-899	Sick leave benefit	Health-related work absenteeism	Health-related work absenteeism	Reduced workability	-	
740 743-748	Unemployed awaiting flexible job					
750 753-758 760 763-768 791 792	Rehabilitation					

810 813-819 784	Vocational rehabilitation program					
785 870 873-879	Workability clarification					
622 781 783 797	Early retirement pensions	Retirement	Retirement	Retirement	-	-
611, 621, 793	Post-employment retirement					
998	Retirement					
997	Not resident in Denmark	Emigrated	Censored or Death	-	-	-
		End of study				
		Deaths				

SEP, Socioeconomic position; DREAM, The Danish Rational Economic Agent Model (DREAM) database.

[1] Hjollund NH, Larsen FB and Andersen JH. Register-based follow-up of social benefits and other transfer payments: accuracy and degree of completeness in a Danish interdepartmental administrative database compared with a population-based survey. Scandinavian journal of public health. 2007; 35: 497-502.

Figure S1 Median (interquartile range) age by categories of multimorbidity and socioeconomic position

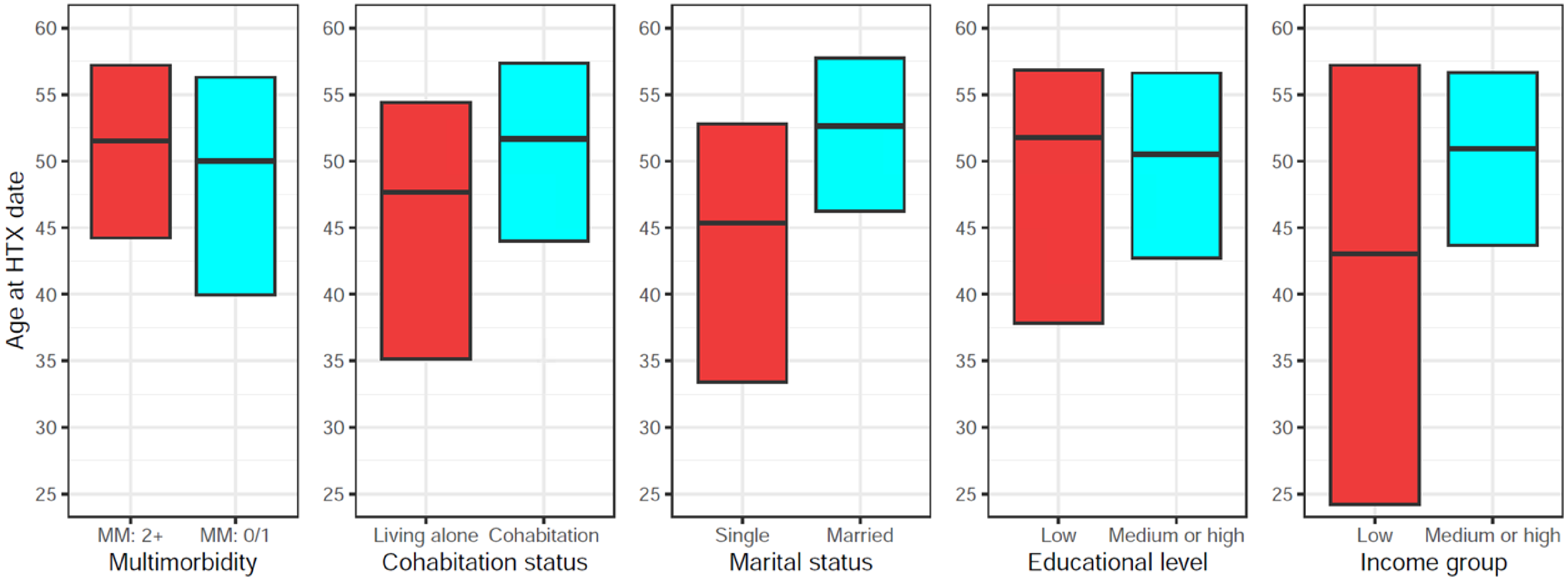

Supplement: Supplementary file 1 [file DataSheet1.PDF]
